# Validity of digital measurement of visual acuity and contrast sensitivity in Parkinson’s disease

**DOI:** 10.1186/s44247-025-00195-z

**Published:** 2025-10-01

**Authors:** Julia Das, Gillian Barry, Rodrigo Vitório, Richard Walker, Yunus Celik, Claire McDonald, Rosie Morris, Samuel Stuart

**Affiliations:** 1 Department of Sport, Exercise & Rehabilitation, Northumbria University, Newcastle upon Tyne, UK; 2 Northumbria Healthcare NHS Foundation Trust, North Tyneside General Hospital, North Shields, UK; 3 Department of Computer and Information Sciences, Northumbria University, Newcastle upon Tyne, UK; 4 Gateshead Health NHS Foundation Trust, Gateshead, UK; 5 Department of Neurology, Oregon Health & Science University, Portland, OR USA

**Keywords:** Visual function, Visual acuity, Contrast sensitivity, Parkinson’s disease, Computerised assessment

## Abstract

**Purpose:**

The Senaptec Sensory Station (SSS) is a device that can measure visual acuity (VA) and contrast sensitivity (CS), but validity has not been established in clinical populations. Therefore, we examined analytical and initial clinical validation of VA and CS measured via the SSS in Parkinson’s disease (PD).

**Methods:**

SSS was used in 34 PD (aged 69.6 (SD = 9.4) years old) to measure VA (via visual clarity outcome) and CS (via SSS-CS6 and SSS-CS18 outcomes). Analytical validation was performed through comparison to reference VA and CS eye-charts (ETDRS VA, MARS CS), and clinical validation was performed through correlation with clinical measures.

**Results:**

Difference in VA LogMar score between the reference eye chart and the SSS was − 0.03 (0.23); e.g., approximately a single letter on the eye chart. There was moderate agreement between the SSS and eye chart VA measurement in PD (ICC = 0.42). Additionally, there was moderate correlation between SSS and eye chart (0.8 cpd) CS measurement in PD, specifically for SSS-CS6 (6 cpd) (*r* = 0.46). However, there was poor correlation between eye chart and SSS-CS18 (18 cpd). SSS VA and CS measures correlated moderately with cognitive function, disease duration and severity, providing clinical validation evidence.

**Conclusion:**

VA and CS can be measured with SSS in PD with moderate comparability to reference eye charts (dependent on cpd for CS), and digital outcomes may provide clinically meaningful outcomes to use in PD trials. A range of human, technological and protocol factors may impact validity of VA and CS measurement via SSS, which should be further examined in future studies.

## Introduction

Visual disorders and retinal abnormalities are often present in Parkinson’s disease (PD) from an early stage, with increasing recognition that visual dysfunctions may occur at a prodromal premotor stage [1, 2]. Studies suggest that visual dysfunction may be a potential biomarker for detecting individuals at an early stage of PD, as well as contributing to differential diagnoses [3–5]. Visual deficits have been shown in basic visual functions, such as visual acuity (VA) and contrast sensitivity (CS), as well as difficulties in higher level (cortical, cognitive) visual processing [6]. Retinal dopamine depletion and decreased dopaminergic innervation of the visual cortex are thought to be among the causes of visual disturbances in PD [7, 8]. Visual deficits have been linked to motor and other non-motor symptoms, such as postural instability [9], impaired driving [10], and increased risk of hallucinations [11], which can cause people with PD to reduce their social and physical activities. In turn, deficits in visual function adversely impact quality of life in people with PD [12]. Timely recognition of visual impairment in PD is therefore important because it allows early and tailored interventions to be established to promote greater independence and improved quality of life [4, 13].

Given the clinical significance and functional implications of visual impairments in PD, there is an increasing demand for reliable and comprehensive methods of assessing visual function within research and clinical practice. The measurement of basic visual functions are traditionally performed using standardised eye charts composed of high contrast black targets (i.e., optotypes such as letters) presented on a white background. However, currently available eye charts are typically limited in their spatial frequencies and resolutions that they can test or present, and can be time consuming when measuring full basic visual functions with those that have visual and cognitive deficits [14]. Alternatively, the rapid development of computer-based systems provides new possibilities in examination of basic visual functions [15], which can provide objective and potentially more granular outcome measures.

The Senaptec Sensory Station (SSS) is a computerised system that can examine basic visual functions of VA and CS through use of a screen and tablet (with underlying algorithms to test a wide range of spatial frequencies and resolutions) [16]. To date, the SSS has been reviewed for test–retest reliability within healthy adults [17, 18], and demonstrated reliable performance on assessments of VA and CS [19]. However, the validity of the SSS VA and CS measurements compared to gold-standard references (traditional eye charts) has not been conducted in people with PD.

This study aimed to (1) assess the analytical validity of visual acuity and contrast sensitivity measured with the SSS against the gold standard reference eye-charts in people with PD; and (2) assess the clinical validity of SSS measured visual acuity and contrast sensitivity through relationship with traditional clinically relevant outcome measures in PD.

## Methods

### Study design

Baseline data from a subset of participants in a pilot randomised controlled trial investigating visual training interventions in PD were used in this cross-sectional analysis [20]. Standardised chart procedures were used to assess VA and CS. All tests were administered binocularly while wearing any habitual correction, if available. This approach has been used in previous studies [21] and was chosen in order to best simulate day-to-day visual function [22]. VA and CS were also assessed as part of a battery of visual tests on the SSS. Participants completed these visual tests as part of a general protocol that included assessments of clinical status, motor performance, cognitive ability and visual function, as well as self-report questionnaires about their mood, quality of life and attitude towards falling (described in Das et al. (2022) [20]).

### Participants

People with PD were recruited through local Movement Disorders clinics, the Parkinson’s UK Research Support Network and the NHR-CPMS Dementias and Neurodegenerative Diseases Research Network in the north-east of England as part of a pilot randomised controlled trial [20]. Inclusion criteria for all participants were: diagnosis of idiopathic PD according to a Movement Disorder specialist; aged > 50 years; normal or corrected-to-normal vision (< 18/6 on the Snellen VA); non-demented cognitive status (≥ 21 on the Montreal cognitive assessment (MoCA) [23]; living independently; able to stand and walk without assistance from another person. Participants were excluded if they had any medical conditions that could interfere with the study safety and conduct, such as psychiatric co-morbidity, unstable cardiovascular disease and neurological conditions other than PD.

### Equipment

#### Visual acuity eye chart

The Early Treatment Diabetic Retinopathy Study (ETDRS) chart is the current gold standard for assessing VA in the UK, as recommended by the International Council of Ophthalmology [24, 25]. The test task requires the use of letters of equal legibility, the same number of letters on each row and uniform between-letter and between-row spacing. The test letters reduce in size from one line to the next in a constant ratio. Errors were recorded on a pre-set score sheet and testing was terminated if participants either made four consecutive errors on a line or read the final letter. The ETDRS chart score was converted to logMAR (logarithm of the minimal angle of resolution) units via the following formula:

LogMar VA = (score of the line before termination)– (0.02 x number of errors) + (0.02 x correct answers in the terminal line).

#### Contrast sensitivity eye chart

The Mars Letter Contrast Sensitivity chart is a development of the Pelli-Robson chart, offering several advantages, including smaller size, improved durability, and ease of use. It is a more convenient assessment of CS in practice, shows excellent agreement with the Pelli-Robson test and has similar repeatability [26]. The contrast range is from 0.04 to 1.92 log units, with each letter representing an increment of 0.04 log units [27]. It presents letters in declining contrast across and down the chart, which captures CS at 0.8 cycles per degree (cpd). The test stops when two consecutive errors are made. The score is the log CS of the final correct letter, minus 0.04 for any errors before that [28]:

LogCS = (score of final correct letter before termination)– 0.04 x number of errors prior to stopping.

#### Senaptec sensory station

**Fig. 1:**
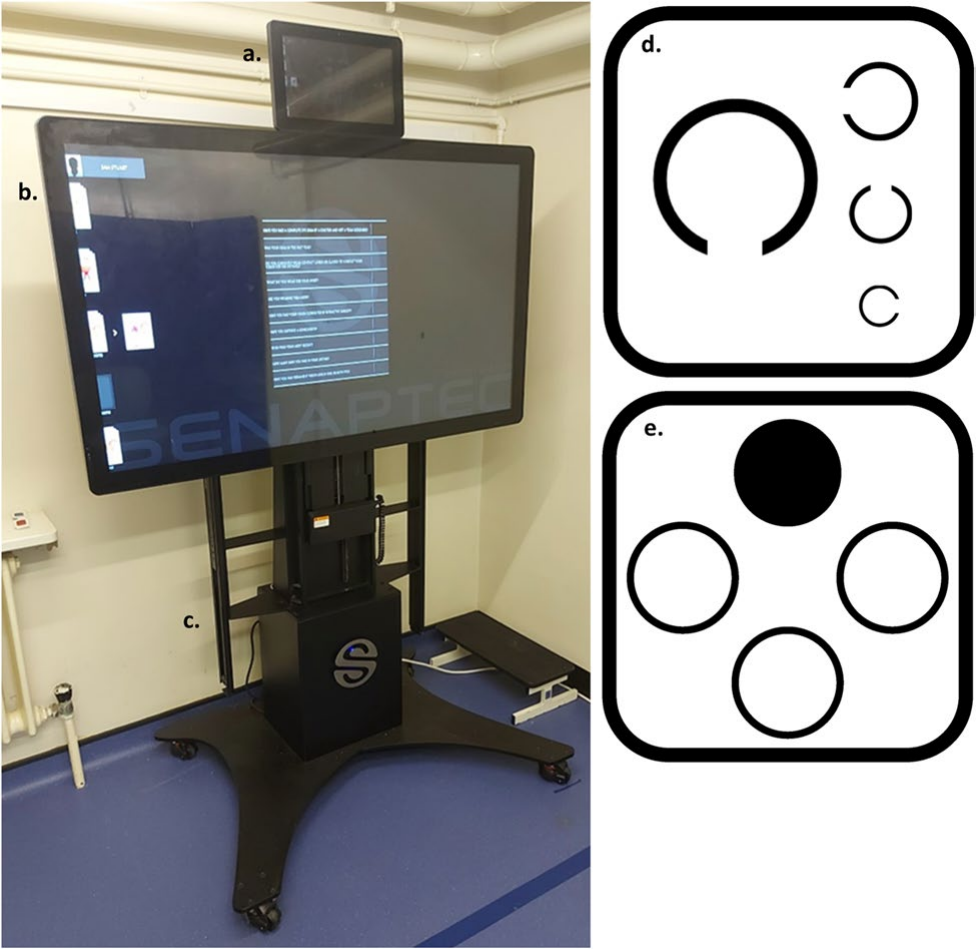
Senaptec Sensory Station; **a**. 13.3-inch tablet; **b**. 55-inch high definition touch screen; **c**. Height adjustable pedestal; **d.** Representation of the Senaptec Sensory Station visual display using Landolt C (visual acuity); **e**. Representation of the Senaptec Sensory Station visual display using contrasting circles (contrast sensitivity)

The SSS is an interactive touch screen device consisting of a computer that controls two LCD monitors, one large 55-inch high definition touch screen and one 13.3-inch tablet, which are mounted on a height adjustable pedestal (Fig. 1). It consists of a battery of visuo-cognitive tests that we have described elsewhere [29]. The tasks measuring VA and CS were presented on the smaller 13.3-inch display monitor, with participants standing at a distance of 10 ft (3 m) away from the Station and responding via a handheld smartphone device that connected to the SSS via Bluetooth. Stimulus presentation and final thresholds were determined on a staircase procedure, in which the difficulty level of the presented stimuli was adjusted in accordance with the performance of the participant. From an initial pre-set starting level (depending on the task), the stimulus was presented at a more difficult level following each correct response, or an easier level if an incorrect response was made. The staircase ended once two adjacent levels each recorded two correct and two incorrect responses. The highest level with two correct responses was defined as the sensitivity threshold for that task [19].

#### Visual clarity

The visual clarity task assessed VA through the minimum detectable spatial resolution for a non-moving, single object. The image consisted of a ring with a break at the top, bottom, left or right with 45 degree positions in between, also known as the “Landolt C” [30] (Fig. 1d). The image was displayed in the centre of the screen and participants were asked to swipe the screen of the smartphone in the direction of the ring break, whilst standing at a distance of 10 feet from the screen. The image gets progressively smaller with every correct swipe, until a visual clarity score is determined.

#### Contrast sensitivity

The CS task assessed the minimum contrast level to distinguish lightness and darkness. Four black circles were presented in a diamond configuration on a light grey background, with one of the circles randomly containing a pattern of concentric rings (Fig. 1e). Participants were asked to swipe the screen of the smartphone in the direction of the circle with the pattern. Stimuli were adjusted following the staircase procedure described above. CS was measured binocularly at 6 and 18 cycles per degree (cpd), and log transformed, with larger values indicating better CS [19].

### Protocol

All participants underwent binocular visual testing using both the standardised eye charts and the SSS on the same day in the Clinical Gait Laboratory at Coach Lane Campus, Northumbria University. The visual tests formed part of the larger battery of demographic, clinical, cognitive and motor performance assessments undertaken for the purposes of a pilot randomised controlled trial described in Das et al. [20]. All tests were performed under the same best corrected visual conditions and in the same testing sequence; logMAR, logCS, SSS-VC, SSS-CS. Artificial lighting was used to illuminate the room (allowing for an average of 80–120 cd/m^2^ luminance across eye charts) and black out screens were used to reduce glare on the SSS screens from outside light sources.

### Statistical analysis

Statistical analysis was performed using SPSS 28.0 (IBM SPSS Inc., 2021). Demographic characteristics were calculated as means and standard deviations (SD). Data were inspected through visual analysis of boxplots and by the Shapiro–Wilk test for normality.

*Visual acuity*: For analytical validation, we compared the VA reference measure (VA ETDRS eye chart, logMAR) to the SSS visual clarity outcome (LogMAR) we conducted intra-class correlation coefficients (ICC) and Bland-Altman plots (with limits of agreement, 95%) to assess the absolute agreement between outcomes [31], as the tests measure VA on the same LogMAR scale. ICC values were classified based on research conducted by Koo and Li [26, 32] and were as follows; Excellent (> 0.90), good (0.75–0.89), moderate (0.50–0.74), and poor (< 0.50). Mean differences and t-test of differences are also reported.

*Contrast sensitivity*: For analytical validation, we compared the CS reference measure (CS MARS eye chart, 0.8 cpd LogCS) to the SSS CS outcomes at 6 cpd and 18 cpd (LogCS). We performed Pearson’s correlations with scatter plots as CS was tested at different cycles per degree (0.8 cpd vs. 6 or 18 cpd) which limits direct comparison. Pearson correlation values were classified as poor (value lies below ± 0.29), moderate (value lies between ± 0.30 and ± 0.49), and large (value lies between ± 0.50 and ± 1) [33].

To examine the clinical validity of the SSS VA and CS test outcomes we performed Pearson’s correlations between SSS-VC, SSS-CS6, SSS-CS18 and clinically relevant outcome measures including demographic (disease duration), motor (UPDRS III, MiniBEST), and cognitive (MoCA) tests. The same interpretation of correlations as above was used.

## Results

### Participants

Data from 34 participants (mean age (SD): 69.6 (9.4) years; 29 male, 5 female; height: 169.7 (8.6) cm; mass: 83.66 (14.0) kg) were included in this study. Participants had an average disease duration of 6.5 (6.1) years, an average Movement Disorders Society-Unified Parkinson’s Disease Rating Scale III of 33.4 (16.4) with the majority of participants categorized as Hoehn & Yahr stage II (H&Y stage I: *n* = 7 (21%); stage II: *n* = 17 (50%); stage III *n* = 10 (29%). Thirteen (38%) participants reported freezing of gait.

### Analytical validation

**Fig. 2:**
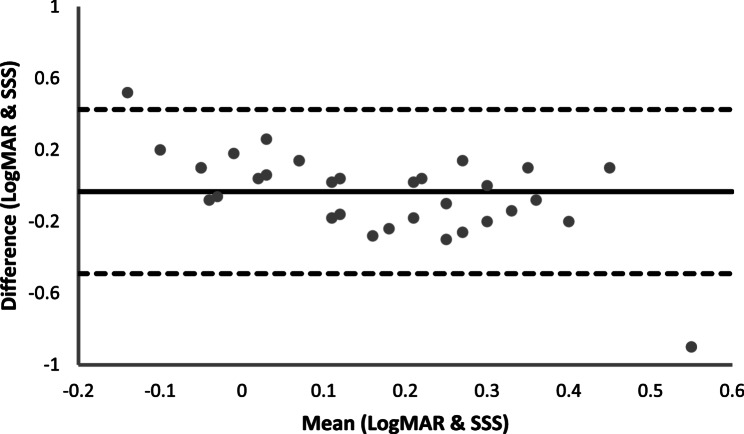
Bland Altman plots displaying agreement between standard chart and SSS-based test for measuring visual acuity

VA and CS obtained from the standard charts and SSS-based tests are summarised in Table 1. The difference between the VA tests (standard– SSS) was − 0.03 (SD = 0.23) logMAR (approximately a single letter on the ETDRS eye chart). There was a significant moderate correlation between the reference eye-chart and SSS-based test for VA (*r* = 0.42, *p* = 0.014). The Bland–Altman plots for VA are shown in Fig. 2, with a moderate ICC (ICC = 0.51, Table 1) and poor limit of agreement (LoA95%=0.58, Table 1) found for comparison between reference eye-chart and SSS-based VA measurement.

**Table 1:** VA and CS obtained from the standard eye charts and SSS-based tests

	Eye Chart Reference Test Mean (SD)		SSS Test Mean (SD)	Difference Mean (SD)	*p*	ICC (Lower bound, Upper bound)	*p*	LoA95%	Pearson *r*	Pearson *p*
**ETDRS LogVA (LogMar)**	0.14 (0.13)	**SSS-VC (LogMar)**	0.18 (0.26)	−0.03 (0.23)	0.417	0.51 (0.02, 0.76)	0.022	0.58	0.42	0.014
**MARS logCS (0.8cpd)**	1.46 (0.16)	**SSS-CS6 (6cpd)**	1.53 (0.38)	-	-	-	-	-	0.45	0.006
		**SSS-CS18 (18cpd)**	0.77 (0.32)	-	-	-	-	-	0.24	0.168

**Fig. 3:**
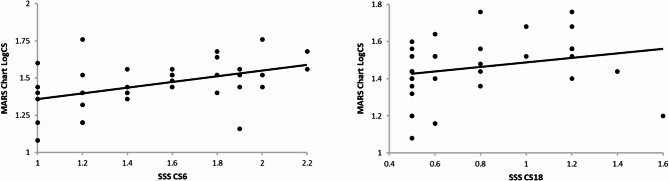
Scatter plots for contrast sensitivity

The CS eye chart and SSS-based tests were on different cycles per degree (i.e., eye chart was 0.8 cpd, compared to 6 and 18 cpd from the SSS) limiting direct comparison of scores (i.e., unable to perform score difference assessment), but there was still a significant moderate correlation between the reference CS eye chart and CS 6 cpd (*r* = 0.45, *p* = 0.006), but not for CS 18 cpd which had a poor relationship with CS eye chart score (*r* = 0.24, *p* = 0.168), (Table 1; Fig. 3).

### Clinical validation

Table 2 shows the correlations between the Eye-chart or SSS outcomes for VA and CS with clinically relevant measures of disease duration, MoCA, UPDRS III and MiniBEST. There were poor to moderate relationships between the digital SSS and traditional clinically relevant measures. Moderate relationships were shown for SSS-VC (*r* = 0.46, *p* = 0.01), SSS-CS6 (*r*=−0.39, *p* = 0.02) and SSS-CS18 (*r*=−0.32, *p* = 0.07) with disease duration, which was better than the poor relationship seen between the standard eye-charts and disease duration (Table 2). The SSS-CS6 (*r*=−0.43, *p* = 0.01) and SSS-CS18 (*r*=−0.46, *p* = 0.01) also related to the UPDRS-III, which was similar to relationships between the eye charts and UPDRS-III (Table 2). Additionally, SSS-VC (*r*=−0.34, *p* = 0.05) and SSS-CS6 (*r* = 0.30, *p* = 0.08) correlated with the MiniBEST, but eye-chart measures had poor correlation with the MiniBEST (Table 2). Interestingly, the MoCA had moderate correlation to VA and CS measured via traditional eye charts (*r*=−0.39, *p* = 0.01, *r* = 0.51, *p* = 0.00, respectively), but only SSS-VC had similar moderate correlation with MoCA (*p*=−0.35, *p* = 0.04) with SSS CS outcomes having poor relation to MoCA (Table 2).This highlighted that worse VA (higher score) related to increased disease duration, and worse CS (lower score) related to worse disease severity (UPDRS III) in PD.

**Table 2:** Correlations between VA and CS scores and other clinically relevant outcomes

Pearson (*r* (*p*))		Disease Duration	MoCA	UPDRS III	MiniBEST
Eye Charts	VA	0.17 (0.32)	−0.39 (0.01)	0.31 (0.06)	−0.21 (0.20)
	CS	−0.28 (0.08)	0.51 (0.00)	−0.44 (0.01)	0.24 (0.14)
Senaptec	SSS-VC	0.46 (0.01)	−0.35 (0.04)	0.28 (0.10)	−0.34 (0.05)
	SSS-CS6	−0.39 (0.02)	0.23 (0.19)	−0.43 (0.01)	0.30 (0.08)
	SSS-CS18	−0.32 (0.07)	0.15 (0.39)	−0.46 (0.01)	0.28 (0.11)

## Discussion

To our knowledge, this is the first study to investigate the analytical and clinical validity of the SSS measurement of VA and CS in people with PD. Results provide evidence that suggest the SSS can measure VA with moderate comparison, and CS with poor-moderate comparison, to established reference methods in PD, and the validity and reliability may be influenced by different factors. Findings contribute to the development of digital visual function assessment in clinical neurological populations.

### Analytical validity

The mean score from the reference eye chart measure was slightly worse (higher) than the SSS test of VA, which was in keeping with a previous similar study in older adults [21]. The average difference between the reference measure and the digital SSS VA measure was only approximately the equivalent of one letter on the eye chart (LogMar 0.03). In comparison, participants tended to achieve better CS values on the SSS than on the reference eye chart, which has been shown in previous studies [21, 34].

Our analytical validation results showed that VA and CS can be measured by the SSS in people with PD with outcomes comparable to the traditional reference eye-charts. Specifically, we showed moderate relationship between measurement methods. However, our comparison of VA and CS was limited due to the different distances measured for VA (4 m on ETDRS and 3 m on SSS) and cpd used for CS outcome measures. The comparison between MARS CS eye chart score and SSS-CS18 score was particularly impacted by the difference in cpd, with only a poor relationship between these measurement methods. While we would expect that CS would correlate across different cpd (i.e., if a participant has good CS at one cpd level they would potentially have good GS at other levels), the larger the difference in the cpd the less the results compared across eye chart and digital measures.

### Challenges in analytical validation

Prior studies that have examined tablet-based vision tests in small samples of adults and have reported better agreement with reference tests than found in the current study [34, 35]. Comparison of digital SSS VA and CS to eye chart measures is influenced by a number of technological, human and protocol factors, which collectively impact the reliability and effectiveness of the analytical validation.

#### Technological factors

Accurate scoring of VA and CS via digital technology may be difficult due to the use of screen-based devices (i.e., the SSS) that may suffer from glare (unlike standard eye-charts), which is supported by previous findings that have shown poorer acuity compared to traditional eye-charts due to screen glare [36]. While precautions were taken to control screen glare within this study (e.g., blocking out natural light and use of artificial lighting) it may still have influenced results.

The visual presentation (e.g., staircase method) and scoring methodology of the digital technology is inherently different from traditional eye charts, with a potentially wider range and more repetitions of different levels until a sensitivity threshold is determined by the underlying algorithm. Additionally, the difference between reading a letter from the standardised charts (i.e., reading each line at each level; but all presented at once) compared to determining the direction of the Landolt C/ring presentations within the SSS (i.e., a C/ring presented in the centre of vision) may limit direct comparison, as the methods used by each technology involve different eye movement/function and cognitive resources that may be impacted by PD.

#### Human factors

Responses on the VA and CS testing via SSS were likely impacted by motor impairment in PD, with the requirement to swipe the screen of the smartphone to register a response to the digital display. Motor symptoms, such as tremor and reduced manual dexterity, impacted the ability for some participants to hold the smartphone response device sufficiently steady with one hand to allow accurate swiping of the screen which may have resulted in response errors. Similarly, speech is commonly impaired in PD from an early stage [37] and may also have impacted eye-chart response. Therefore, future studies should control for PD symptoms that may influence stimulus response.

Familiarity with the standardised eye-charts (i.e., prior experience in clinical settings) over the digital measures may have led to greater error rate on the digital SSS responses, as we subjectively observed participants required further assistance to understand and respond to visual stimuli on the screen compared to traditional eye-charts. Furthermore, when using eye-charts participants are able to verbally correct their answers if they were aware of a mistake during the testing procedure, whereas in its current format, the SSS testing sequence did not allow for withdrawal of an erroneous response if a participant produced an unintentional mis-swipe on the screen.

#### Protocol factors

Selection of an appropriate reference methodology/technology for analytical validation testing of the SSS VA and CS digital measures was limited to traditional clinically implemented eye-charts. While there was relationship between the reference eye-charts and SSS for the measurement of VA and CS, previous studies suggest that there may be inherent error in the reference eye-chart measurements of visual function that may have impacted findings [38, 39]. Specifically, previous work has shown that the traditional eye-chart methods are prone to observer bias (e.g., a researcher may influence participant response) and due to the standardisation of the eye-charts they can be memorised with repeated testing [40]. In comparison, the SSS has an absence of observer bias due to not relying on a researcher (or clinician) to score the test and the SSS digital random-image generation avoids the memorisation effects associated with standard eye-charts [41, 42]. However, in future comparison to other measures of VA and CS (i.e., various eye-charts or other digital tests) both monocularly and binocularly, or potentially other ophthalmological testing, may improve analytical validation protocols for digital methodologies.

### Clinical validity

Clinical validity of VA and CS measurement via SSS in PD was shown through correlation with traditional clinically relevant measures, in line with the BEST evidentiary framework [43] and V3 validation framework [44]. Despite increasing research on visual changes in PD, studies examining the correlation of visual function with PD features such as motor severity remain scarce [45, 46]. Our findings showed that disease duration and severity (UPDRS III), and cognitive function were moderately related to SSS measures of VA and CS in PD, which was similar to standard eye-chart measures and highlighted that worse motor or cognitive function and disease related to poorer visual function in PD. However, relationship between visual function assessment and other clinical assessments was not universally consistent across traditional eye-chart or SSS VA and CS measures. For example, better CS measured via eye chart related to better cognition (MoCA), but this was not found within the SSS CS outcomes, and alternatively longer disease duration related to SSS VA and CS measures but not eye-chart measures. While this study was not powered to provide definitive results on whether these digital outcome measures from the SSS can be used as biomarkers of disease progression or therapeutic intervention, these initial relationships with clinically meaningful outcomes are promising. Future studies are required to further investigate the nuances between VA and CS measurement with eye-charts or digital tools in PD to examine the specific clinical relevance of digital measurement.

## Conclusion

Digital visual acuity and contrast sensitivity assessment via Senaptec Sensory Station can be measured with moderate analytical validity (dependent on cpd for CS) in PD compared to reference measures. Digital visual acuity and contrast sensitivity measures related to cognitive function, disease duration and severity in PD, which showed their potential clinical meaningfulness as outcome measures in future trials. Future studies with larger sample sizes are needed to substantiate findings and to evaluate the test-retest reliability while controlling for technological, human and protocol factors that could impact results.

## Data Availability

Data is available upon request to the principal investigator (SS).
